# Dexamethasone diminishes the pro-inflammatory and cytotoxic effects of amyloid β-protein in cerebrovascular smooth muscle cells

**DOI:** 10.1186/1742-2094-3-18

**Published:** 2006-08-03

**Authors:** Mary Lou Previti, Weibing Zhang, William E Van Nostrand

**Affiliations:** 1 Department of Medicine, Health Sciences Center, Stony Brook University, Stony Brook, NY 11794-8153, USA

## Abstract

**Background:**

Cerebrovascular deposition of fibrillar amyloid β-protein (Aβ), a condition known as cerebral amyloid angiopathy (CAA), is a prominent pathological feature of Alzheimer's disease (AD) and related disorders. Accumulation of cerebral vascular fibrillar Aβ is implicated in promoting local neuroinflammation, causes marked degeneration of smooth muscle cells, and can lead to loss of vessel wall integrity with hemorrhage. However, the relationship between cerebral vascular fibrillar Aβ-induced inflammatory responses and localized cytotoxicity in the vessel wall remains unclear.

Steroidal-based anti-inflammatory agents, such as dexamethasone, have been reported to reduce neuroinflammation and hemorrhage associated with CAA. Nevertheless, the basis for the beneficial effects of steroidal anti-inflammatory drug treatment with respect to local inflammation and hemorrhage in CAA is unknown. The cultured human cerebrovascular smooth muscle (HCSM) cell system is a useful *in vitro *model to study the pathogenic effects of Aβ in CAA. To examine the possibility that dexamethasone may influence CAA-induced cellular pathology, we investigated the effect of this anti-inflammatory agent on inflammatory and cytotoxic responses to Aβ by HCSM cells.

**Methods:**

Primary cultures of HCSM cells were treated with or without pathogenic Aβ in the presence or absence of the steroidal anti-inflammatory agent dexamethasone or the non-steroidal anti-inflammatory drugs indomethacin or ibuprofen. Cell viability was measured using a fluorescent live cell/dead cell assay. Quantitative immunoblotting was performed to determine the amount of cell surface Aβ and amyloid β-protein precursor (AβPP) accumulation and loss of vascular smooth cell α actin. To assess the extent of inflammation secreted interleukin-6 (IL-6) levels were measured by ELISA and active matrix metalloproteinase-2 (MMP-2) levels were evaluated by gelatin zymography.

**Results:**

Pathogenic Aβ-induced HCSM cell death was markedly reduced by dexamethasone but was unaffected by ibuprofen or indomethacin. Dexamethasone had no effect on the initial pathogenic effects of Aβ including HCSM cell surface binding, cell surface fibril-like assembly, and accumulation of cell surface AβPP. However, later stage pathological consequences of Aβ treatment associated with inflammation and cell degeneration including increased levels of IL-6, activation of MMP-2, and loss of HCSM α actin were significantly diminished by dexamethasone but not by indomethacin or ibuprofen.

**Conclusion:**

Our results suggest that although dexamethasone has no appreciable consequence on HCSM cell surface fibrillar Aβ accumulation it effectively reduces the subsequent pathologic responses including elevated levels of IL-6, MMP-2 activation, and depletion of HCSM α actin. Dexamethasone, unlike indomethacin or ibuprofen, may diminish these pathological processes that likely contribute to inflammation and loss of vessel wall integrity leading to hemorrhage in CAA.

## Background

Deposition of the amyloid β-protein (Aβ) in brain is a prominent pathological feature of Alzheimer's disease (AD) and a number of related disorders [1,2]. Aβ is a 39–43 amino acid peptide that exhibits a high propensity to self-assemble into β sheet-containing oligomeric forms and fibrils. Aβ peptides are proteolytically derived from a large type I integral membrane precursor protein, termed the amyloid β-protein precursor (AβPP) through sequential cleavage by β- and γ-secretase activities [1,2]. Cerebral parenchymal Aβ deposition can occur as diffuse plaques, with little or no surrounding neuropathology, or as dense, fibrillar plaques that are associated with dystrophic neurons, neurofibrillary tangles, and neuroinflammation [1,2]. In addition to plaques in the brain parenchyma, another prominent site of extracellular Aβ deposition is within and along primarily small and medium-sized arteries and arterioles of the cerebral cortex and leptomeninges and in the cerebral microvasculature, a condition known as cerebral Aβ angiopathy (CAA) [3,4]. In contrast to the dichotomous nature of diffuse or fibrillar parenchymal plaques, CAA largely exists as fibrillar Aβ deposits [3-5]. Accumulation of cerebral vascular fibrillar Aβ has been shown to cause marked degeneration and cell death of smooth muscle cells and pericytes in affected larger cerebral vessels and in cerebral microvessels, respectively [4-7]. Recent findings have implicated cerebral microvascular Aβ deposition in promoting neuroinflammation and dementia in AD [8-11].

In addition to the prominent CAA that is found in AD and in spontaneous cases of this condition, several monogenic, familial forms of CAA exist that result from mutations that reside within the Aβ peptide sequence of AβPP gene [12-15]. The most recognized example of familial CAA is the Dutch-type disorder that causes early and severe cerebral vascular amyloid deposition [16]. Dutch-type CAA results from a E22Q substitution within the Aβ peptide [12]. Pathologically, this disorder is characterized by vascular amyloid-associated neuroinflammation and recurrent, and often fatal, intracerebral hemorrhages at mid-life [17-19].

Neuroinflammation associated with CAA is a chronic process that involves well-recognized cellular mediators including reactive astrocytes and activated microglia, as well as inflammatory cytokines and chemokines [20,21]. Additionally, cerebral vascular smooth muscle cells and pericytes, which degenerate in the presence of accumulated amyloid, may directly participate in the inflammatory process contributing to cognitive decline, cerebral vessel wall degeneration, and hemorrhage [3-7]. Recent case reports have emerged demonstrating that steroidal anti-inflammatory treatments aimed at reducing CAA-induced neuroinflammation and associated pathology in afflicted individuals have improved the cognitive deficits and recurrent hemorrhage associated with this particular condition [22-25]. However, the basis for the success of steroidal anti-inflammatory dugs in treating the pathological consequences of CAA remains unknown.

To investigate how steroidal anti-inflammatory agents may suppress CAA-related inflammation and vessel wall pathology, we determined the effects of dexamethasone on Aβ-induced pathologic responses in human cerebrovascular smooth muscle (HCSM) cells, a well-established *in vitro *model of CAA. Our results indicate that although Aβ accumulation and fibril formation on the surface of HCSM cells was not affected, dexamethasone effectively reduced the levels of IL-6, MMP-2 activation, and loss of smooth muscle α actin, processes that precede HCSM cell death and likely contribute to inflammation and loss of vessel wall integrity in CAA.

## Methods

### Materials

Dulbecco's modified Eagle medium (DMEM) and fetal bovine serum was obtained from Gibco-BRL (Grand Island, NY). Dexamethasone, ibuprofen, and indomethacin were obtained from Sigma (St. Louis, MO) and resuspended as 1 mM stocks in ethanol. The Live/Dead *Euko *Light Viability/Cytotoxicity Kit was obtained from Molecular Probes (Eugene, OR). Human AβPP-specific monoclonal antibody (mAb) P2-1 was prepared as described [26]. The anti-human Aβ mAb6E10 was from Signet Laboratories (Dedham, MA) and the anti-smooth muscle cell α actin mAb1A4 was obtained from Sigma (St. Louis, MO). Immunoblotting reagents were from Amersham (Arlington Heights, IL). The human IL-6 ELISA kit was obtained from BioSource (Camarillo, CA).

### Dutch mutant Aβ40 peptide

The E22Q Dutch mutant Aβ40 peptide was synthesized by solid-phase F-moc amino acid substitution and purified by reverse-phase HPLC. The structure of the peptide was verified and the purity determined by electrospray mass spectrometry and amino acid sequencing by automated Edman degradation. Dutch mutant Aβ40 peptide was prepared in hexafluoroisopropanol, resuspended in sterile dimethyl sulfoxide to a concentration of 5 mM, then diluted to 25 μM in DMEM prior to addition to the HCSM cells. Under these conditions, upon resuspension in DMEM the peptide exhibited a nonaggregated structure in the culture medium throughout the six days incubation period used in the experiments.

### Cell culture

HCSM cells were established from meningeal blood vessels obtained at rapid autopsy as described [27]. Three different lines of primary HCSM cell cultures were used in the present experiments. The HCSM cells were cultured in DMEM containing 10% fetal calf serum and antibiotics. The cells were passaged at a 1:4 split ratio and used between passages 4–8 for all experiments. HCSM cells were incubated for six days in the presence or absence of freshly prepared 25 μM Dutch mutant Aβ40. HCSM cells were administered daily with dexamethasone, ibuprofen, or indomethacin to a final concentration of 1 μM. Control HCSM cells received vehicle only. Cells were routinely viewed using an Olympus IX70 phase contrast microscope to monitor cellular degeneration. At the conclusion of the experiments the loss in HCSM cell viability was measured using a fluorescent live cell/dead cell assay as described [28].

### Quantitative immunoblotting

For analysis of cellular proteins, the culture medium was collected and the cells rinsed with phosphate-buffer saline. The cells were then solubilized in a buffer consisting of 50 mM Tris-HCl, 150 mM NaCl, pH 7.5 containing 1% SDS, 5 mM EDTA, and Complete proteinase inhibitor cocktail (Roche, Indianapolis, IN). The cell lysates were centrifuged at 14,000 × *g *for 10 min to remove insoluble material. The protein concentrations of the resulting supernatants were determined using the BCA kit (Pierce, Rockford, IL). The cell lysate samples were stored at -70°C until analysis. Measurement of cell-associated AβPP, Aβ, and smooth muscle cell α actin were performed by quantitative immunoblotting using mAbP2-1, mAb6E10, and mAb1A4, respectively. Purified protein or cell lysate samples were electrophoresed on SDS 10% polyacrylamide gels for AβPP and smooth muscle cell α actin or 10–20% gradient gels for Aβ. The proteins were then electroblotted onto nitrocellulose membranes and the unoccupied sites were blocked overnight with a solution of Tris-buffered saline containing 5.0% nonfat dried milk and 0.05% Tween-20. The membranes were then incubated with the appropriate antibody (1:1000) and, after washing, bound primary antibody was detected with a peroxidase-coupled anti-mouse IgG (1:200). The peroxidase activity on the membranes was detected using Supersignal Dura West (Pierce) and the corresponding bands were quantitated using a VersaDoc Multi-Imager (BioRad Laboratories, Hercules, CA) with the manufacturer's Quantity One software.

### Quantitation of HCSM cell surface Aβ

HCSM cells were grown to near confluency in 24-well tissue culture plates, placed in serum-free culture medium overnight, and then incubated in the absence or presence of 25 μM Dutch mutant Aβ40 with or without dexamethasone in serum-free culture medium for six days. After incubation, the cells were rinsed five times with phosphate-buffered saline and fixed for 30 min at room temperature in 2% paraformaldehyde. The fixed cells were extensively washed with phosphate-buffered saline and then incubated for 15 min with Protein Blocker solution (Research Genetics, Huntsville, AL), rinsed three times with phosphate-buffered saline, and incubated with mAb6E10 (1:1000) overnight at 4°C. The next day the cells were rinsed five times with phosphate-buffered saline and incubated with fluorescein-coupled sheep anti-mouse IgG (1:200) for 1 h at 22°C. The cells were then rinsed five times with phosphate-buffered saline and Aβ immunofluorescence on the HCSM cells was measured at excitation 485 nm and emission 530 nm in a SpectraMax fluorescence plate reader (Molecular Devices, Sunnyvale, CA). Each measurement was performed in triplicate and five fields were scanned for each well.

Alternatively, the fixed HCSM cells were rinsed with phosphate-buffered saline, stained with 0.1% thioflavin T for 10 min at room temperature and rinsed with 80% ethanol three times. Then 250 μl of phosphate-buffered saline was added to each well and Thioflavin T fluorescence was measured at excitation wavelength 440 nm and emission wavelength 485 nm using the SpectraMax fluorescence plate reader. Each measurement was performed in triplicate and five fields were scanned for each well.

### IL-6 ELISA measurements

Conditioned cell culture supernatants were collected from triplicate samples of HCSM cells incubated with or without Dutch mutant Aβ40 in the presence or absence of anti-inflammatory drugs for six days. The samples were centrifuged at 14,000 × *g *to remove any cellular debris. The level of IL-6 in each of the samples was determined using the Ultrasensitive IL-6 ELISA kit as described by the manufacturer (BioSource International Inc., Camarillo, CA).

### Gelatin substrate zymography

Conditioned media samples from HCSM cells treated with or without 25 μM Dutch mutant Aβ40 in the absence and presence of anti-inflammatory drugs were electrophoresed on SDS 8% polyacrylamide gels containing 0.1% gelatin at 100 V for 2 hr at 22°C. The gels were removed and incubated in rinse buffer (50 mM Tris, pH7.5, 200 mM NaCl, 5 mM CaCl_2_, 2.5% Triton X-100) for 3 h with several changes, washed 3 × 10 minutes with ddH_2_O, then incubated in assay buffer (50 mM Tris, pH 7.5, 200 mM NaCl, 5 mM CaCl_2_) overnight at 37°C, washed 3 × 10 minutes with ddH_2_0, stained with 0.25% Coomassie Brilliant Blue R-250 and then destained. Gelatinolytic MMP-2 activity was observed as clear zones of lysis.

### Statistical analysis

Data were analyzed by student's *t *test at *p *< 0.05 significance level.

## Results and discussion

The condition of CAA, and in particular when associated with mutations in Aβ such as in the Dutch-type disorder, is characterized by vascular amyloid-associated neuroinflammation and intracerebral hemorrhage [12-19]. Reactive astrocytes and activated microglia are found adjacent to cerebral vascular fibrillar amyloid deposits and produce a variety of inflammatory mediators including pro-inflammatory cytokines such as IL-1β, IL-6, and tumor necrosis factor-α, as well as chemokines, reactive oxygen species, proteolytic enzymes, and complement proteins [29-32]. Similarly, cerebral vascular smooth muscle cells, which degenerate in the presence of accumulated vascular fibrillar amyloid, may also participate in the inflammatory process contributing to cognitive decline, cerebral vessel wall degeneration, and hemorrhage [17-19]. A significant role for inflammation in the pathology of CAA is supported by recent case reports showing that steroidal-based anti-inflammatory treatments improved cognitive deficits and recurrent hemorrhage associated with cerebral vascular amyloid [22-25].

**Figure 1:**
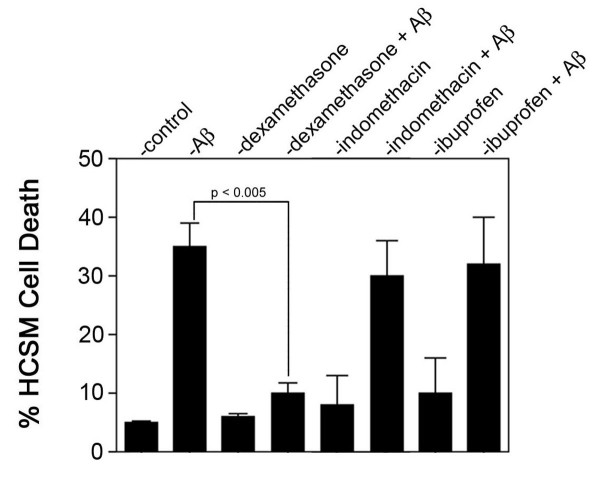
Dexamethasone reduces pathogenic Aβ-induced HCSM cell death. Near confluent cultures of HCSM cells were incubated in the presence or absence of 25 μM Dutch mutant Aβ40 with daily administration of anti-inflammatory drug (1 μM final concentration). After six days the viability of the HCSM cells was determined using a fluorescent live cell/dead cell assay. The data presented are the mean ± S.D. of triplicate wells from three separate experiments.

To begin to understand how steroidal anti-inflammatory drugs may suppress CAA-related neuroinflammation and cellular pathology within the cerebral vessel wall, we determined the effects of dexamethasone and two non-steroidal anti-inflammatory drugs (NSAIDs), ibuprofen and indomethacin, on Aβ-induced toxicity in HCSM cells, a well-established *in vitro *model of CAA. In these studies we used Dutch mutant Aβ40 since we previously showed that this peptide promotes strong pathologic responses in HCSM cells compared with wild-type Aβ peptides [33,34]. Dexamethasone markedly reduced the extent of HCSM cell death caused by Dutch mutant Aβ40 treatment (Figure 1). In contrast, the NSAIDs indomethacin and ibuprofen had no effect on Dutch mutant Aβ40-induced HCSM cell death.

**Figure 2:**
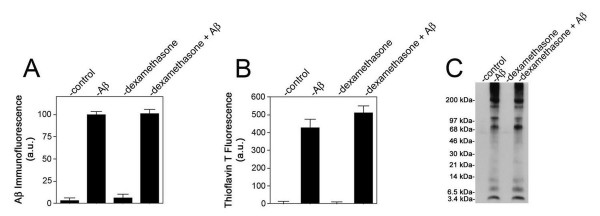
Dexamethasone does not affect pathogenic Aβ accumulation and assembly on the HCSM cell surface. Near confluent cultures of HCSM cells were incubated for six days in the presence or absence of 25 μM Dutch mutant Aβ40 with or without daily administration of dexamethasone (1 μM final concentration). The HCSM cells were extensively washed, then fixed, and cell-surface Aβ was quantitatively measured by immunofluorescence labeling using mAb6E10 (A) or fluorescent thioflavin T binding (B). The data presented are the mean ± S.D. of triplicate wells read in five different fields per well from two to three separate experiments. (C) Alternatively, after incubation the HCSM cells were extensively washed, solubilized, subjected to SDS-PAGE, and subsequently analyzed by immunoblotting using mAb6E10.

**Figure 3:**
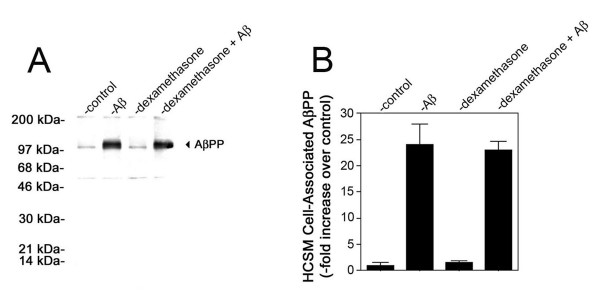
Dexamethasone does not affect cell surface accumulation of AβPP in pathogenic Aβ-treated HCSM cells. Near confluent cultures of HCSM cells were incubated for six days in the presence or absence of 25 μM Dutch mutant Aβ40 with or without daily administration of dexamethasone (1 μM final concentration). The HCSM cells were extensively washed, solubilized, subjected to SDS-PAGE, and subsequently analyzed by quantitative immunoblotting using mAbP2-1. (A) Representative immunoblot and (B) summary of quantitative data. The data are presented as -fold increase in cell-associated AβPP compared to untreated HCSM cells. The data presented are the mean ± S.D. six separate determinations.

Since only the steroidal anti-inflammatory agent dexamethasone blocked pathogenic Aβ-induced HCSM cell death we next determined if this drug disrupts early HCSM cell surface events involved with Aβ toxicity. For example, we previously showed that soluble, unassembled pathogenic forms of Aβ bind to and assemble into fibrillar structures on the surfaces of HCSM cells and that this process is necessary to induce subsequent pathologic responses in the cells [34]. Since dexamethasone blocked HCSM cell death we investigated whether this agent interfered with Aβ accumulation and fibrillar assembly on the cell surface. Quantitative Aβ immunofluorescence measurements showed that dexamethasone did not alter the total amount of Aβ that accumulated on the HCSM cell surface (Figure 2A). Similarly, quantitative thioflavin T fluorescence measurements for HCSM cell surface fibrillar Aβ structures showed no significant difference in the presence of dexamethasone (Figure 2B). Immunoblot analysis of the Aβ that accumulated on the HCSM cell surface revealed a very similar pattern of monomers, dimers, trimers, and particularly, larger massed Aβ structures either in the presence of absence of dexamethasone (Figure 2C). We reported that after the assembly of Aβ fibrils on the HCSM cell surface there is a striking accumulation of cell-associated AβPP [33,34]. This consequence is mediated through its high-affinity binding to the cell surface Aβ fibrils via a domain in the amino terminal region of AβPP [35]. Quantitative immunoblotting revealed that dexamethasone had no demonstrable effect on AβPP accumulation in Dutch mutant Aβ40 treated HCSM cells (Figure 3). Together, these results indicate that dexamethasone does not interfere with the initial pathogenic accumulation and fibrillar assembly of Aβ on the HCSM cell surface nor the subsequent increase in cell surface AβPP that accumulates through binding to the assembled Aβ fibrils. These findings suggest that dexamethasone must target more downstream pathologic events involved with Aβ-mediated inflammation and toxicity in HCSM cells.

**Figure 4:**
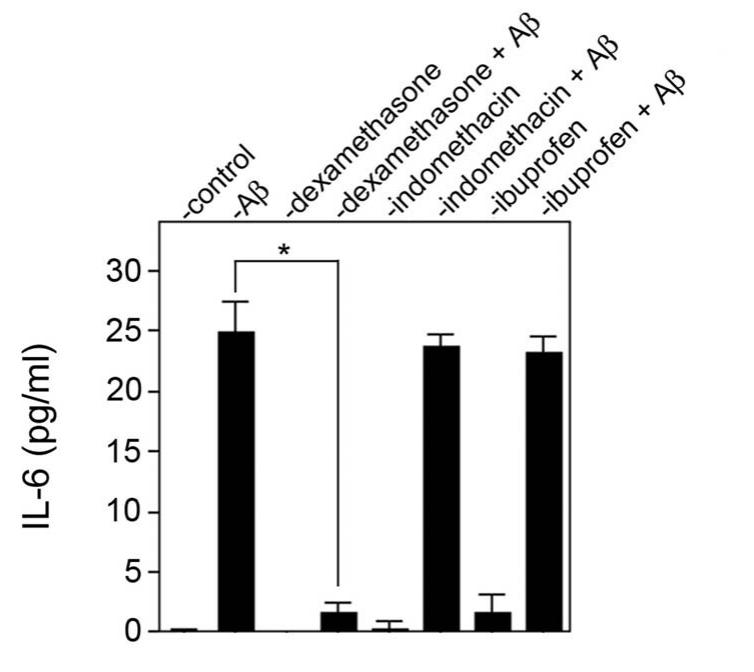
Dexamethasone reduces the levels of IL-6 in pathogenic Aβ-treated HCSM cells. Near confluent cultures of HCSM cells were incubated for six days in the presence or absence of 25 μM Dutch mutant Aβ40 with or without daily administration of anti-inflammatory drug (1 μM final concentration). The cell culture medium was collected and the level of IL-6 present in the medium was measured by ELISA analysis. The data presented are the mean ± S.D. of triplicate samples from two separate experiments.

We next investigated if downstream contributors of vascular amyloid-mediated inflammation, hemorrhage, and cell death are altered by dexamethasone treatment. For example, IL-6 was recently identified as an elevated pro-inflammatory mediator specifically associated with vascular accumulation of fibrillar amyloid in transgenic mice [36]. Although reactive astrocytes and activated microglia, which are strongly associated with fibrillar amyloid in CAA, are known to express IL-6 it is unknown if HCSM cells can also produce this inflammatory cytokine and actively participate in vascular amyloid-mediated inflammation. HCSM cells treated with Dutch mutant Aβ40 exhibited a robust increase in IL-6 expression and, significantly, dexamethasone strongly suppressed the expression of this inflammatory cytokine (Figure 4). In contrast, the NSAIDs indomethacin and ibuprofen had no effect on IL-6 levels released by HCSM cells treated with pathogenic Aβ (Figure 4).

**Figure 5:**
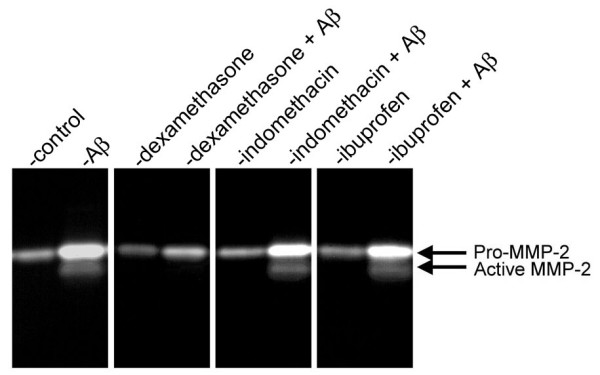
Dexamethasone blocks the activation of MMP-2 in HCSM cells treated with pathogenic Aβ. Near confluent cultures of HCSM cells were incubated for six days in the presence or absence of 25 μM Dutch mutant Aβ40 with or without daily administration of anti-inflammatory drug (1 μM final concentration). The cell culture medium was collected and analyzed by gelatin zymography. Two or three separate experiments were performed in triplicate for each tested condition. A representative zymogram is shown.

Increased expression and activation of matrix metalloproteinase 2 (MMP-2) is associated with neuroinflammation [37]. Furthermore, MMP-2 has been shown to promote opening of the blood-brain barrier and intracerebral hemorrhage by disrupting the extracellular matrix (ECM) [38,39]. In addition to a direct loss of vessel wall integrity, MMP-2 mediated degradation of ECM components may lead to loss of specific ECM-integrin interactions resulting in apoptotic vascular cell death [40]. Recently, we reported that HCSM cells increase their expression and activation of MMP-2 in response to treatment with pathogenic Aβ and that inhibition of MMP-2 activation protected the cells from apoptosis [41]. Similarly, we found that dexamethasone, which reduced HCSM cell death (Figure 1), effectively suppressed increased expression and activation of MMP-2 in response to Dutch mutant Aβ40 (Figure 5). On the other hand, indomethacin and ibuprofen, which did not block HCSM cell death, did not suppress expression and activation of MMP-2 (Figure 5).

**Figure 6:**
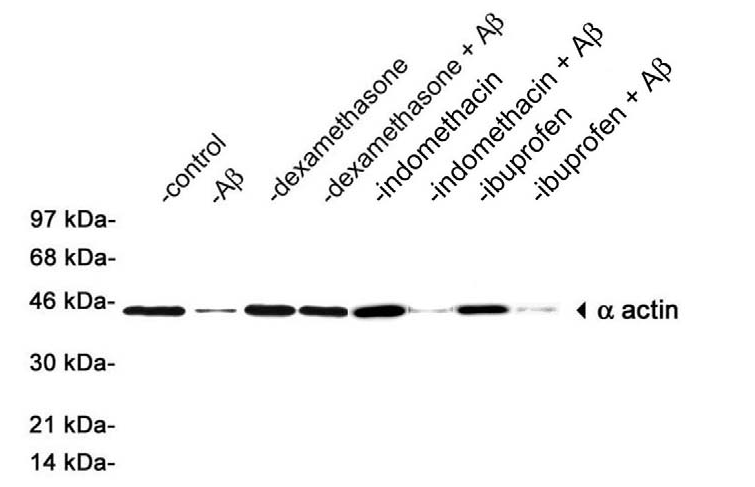
Dexamethasone prevents pathogenic Aβ-induced degradation of HCSM cell α actin. Near confluent cultures of HCSM cells were incubated for six days in the presence or absence of 25 μM Dutch mutant Aβ40 with or without daily administration of anti-inflammatory drug (1 μM final concentration). The HCSM cells were extensively washed, solubilized, subjected to SDS-PAGE, and subsequently analyzed by quantitative immunoblotting using mAb1A4 to smooth muscle cell α actin. Two or three separate experiments were performed in triplicate for each tested condition. A representative immunoblot is shown.

Loss of smooth muscle cell α actin is a prominent consequence of cerebral vascular amyloid accumulation in humans and transgenic mouse models that develop CAA [4-7,36]. This deficit reflects the degeneration and death of smooth muscle cells in the affected vessels, which contributes to loss of vessel wall integrity and hemorrhage. Similarly, HCSM cells treated with pathogenic Aβ also exhibit a striking loss of smooth muscle cell α actin that is a predecessor to apoptotic cell death [28]. Accordingly, HCSM cells treated with pathogenic Dutch mutant Aβ40 showed a ≈90% loss in the levels of smooth muscle cell α actin, a consequence that was essentially prevented in the presence of dexamethasone (Figure 6). In contrast, neither indomethacin nor ibuprofen was capable of preventing loss of smooth muscle cell α actin in HCSM cells treated with Dutch mutant Aβ40 (Figure 6).

There has been much interest in the potential use of anti-inflammatory drugs for the treatment of AD and other Aβ-depositing disorders. This interest arises from epidemiological studies that point to prolonged use of NSAIDs in reducing the risk of AD, delaying the age of onset, and slowing the progression of disease and cognitive impairments [42,43]. Despite the potential for NSAIDs and steroidal anti-inflammatory drugs in the treatment of AD success in slowing disease progression has not been forthcoming in clinical trials [44]. This lack of success may reflect intervention too late in the disease process. On the other hand, recent case reports have emerged demonstrating that steroidal-based anti-inflammatory treatments aimed at reducing CAA-induced neuroinflammation and associated pathology in afflicted individuals have improved cognitive deficits and recurrence of cerebral hemorrhage associated with this particular condition [22-25]. However, the bases for these successes regarding CAA remain unknown. Using our HCSM cell *in vitro *model for CAA we found that although steroidal-based anti-inflammatory treatment had no effect on vascular accumulation and assembly of Aβ it effectively reduced processes of inflammation and cell degeneration, developments that likely contribute to cognitive deficits, loss of cerebral vessel wall integrity, and hemorrhage. Future analysis of these specific deleterious events in animal models and human cases of CAA may yield new avenues for intervening in the pathology of CAA.

## Abbreviations

Aβ, amyloid β-protein; CAA, cerebral amyloid angiopathy; AD, Alzheimer's disease; HCSM, human cerebrovascular smooth muscle; AβPP, amyloid β-protein precursor; IL-6, interleukin-6; MMP-2, matrix metalloproteinase-2; DMEM, Dulbecco's modified Eagle medium; NSAID, non-steroidal anti-inflammatory drug; ECM, extracellular matrix;

## Declaration of competing interests

The author(s) declare that they have no competing interests.

## Authors' contributions

MP participated in experimental design and carried out cytotoxicity experiments, ELISA, and zymography, and data analysis.

WZ carried out cytotoxicity experiments, thioflavin T fluorescence assays, quantitative immunoblotting, and data analysis.

WVN conceived of the study, participated in its design, and helped draft the manuscript.
